# Microencapsulated Multifunctionalized Graphene Oxide Equipped with Chloroquine for Efficient and Sustained siRNA Delivery

**DOI:** 10.1155/2022/5866361

**Published:** 2022-04-06

**Authors:** Rana Imani, Satya Prakash, Hojatollah Vali, John F. Presley, Shahab Faghihi

**Affiliations:** ^1^Department of Biomedical Engineering, Amirkabir University of Technology, Tehran 15875/4413, Iran; ^2^Stem Cell and Regenerative Medicine Group, National Institute of Genetic Engineering and Biotechnology, Tehran 14965/161, Iran; ^3^Biomedical Technology and Cell Therapy Research Laboratory, Department of Biomedical Engineering, Faculty of Medicine, McGill University, Montréal, QC, Canada H3A 2B4; ^4^Department of Anatomy and Cell Biology, McGill University, 3640 University Street, Montréal, QC, Canada H3A 0C7; ^5^Facility for Electron Microscopy Research, McGill University, 3640 University Street, Montréal, QC, Canada H3A 0C7

## Abstract

A multifunctionalized graphene oxide (GO)-based carrier with conjugation of aminated-polyethylene glycol (PEG-diamine), octaarginine (R8), and folic acid (FA), which also contains chloroquine (CQ), a lysosomotropic agent, is introduced. The cellular uptake mechanisms and intracellular targeting of FA-functionalized nanocarriers are examined. The localized releases of CQ and siRNA intracellular delivery are evaluated. Microencapsulation of the nanocarrier complexed with genes in layer-by-layer coating of alginate microbeads is also investigated. The covalently coconjugated FA with PEG and R8 provides a stable formulation with increased cellular uptake compared to FA-free carrier. The CQ-equipped nanocarrier shows a 95% release of CQ at lysosomal pH. The localized release of the drug inside the lysosomes is verified which accelerates the cargo discharge into cytoplasm.

## 1. Introduction

The integration of nanotechnology with gene therapy is a versatile strategy that increases the potential for successful nucleic acid delivery to the desired cells [1]. The cell targeting of gene-nanocarrier complexes is a key factor in the success of gene delivery and in the treatment of genetic-based diseases. Complex instability and degradation of the genetic materials during intracellular trafficking are other challenging obstacles hindering efficient gene transfection. Endocytosis, the major internalization pathway used by most nanocarriers, causes the entrapment of nucleic acids in lysosomes. Several strategies have been developed to overcome lysosomal entrapment and possible degradation of nucleic acid cargos. These strategies are mainly based on destabilization of endosomal and lysosomal membranes [2]. Addition of lysosomotropic compounds in the cell culture media can greatly enhance the transfection efficacy [3] as they usually become protonated at low pH and accelerate the release of genetic materials into the cytoplasm as a result of swelling and rupturing of endosomal compartments. However, such treatments may result in cytotoxicity. Some lysosomotropic agents have been used in combination with nonviral carriers to improve the efficiency of gene delivery. Chloroquine (CQ), known as an antimalarial medication which has been recently introduced as anticancer drug [4], is a lysosomotropic agent that preferentially accumulates in lysosomal compartments [5]. Therefore, it has been often used to enhance transfection efficacy of genes. Although low concentrations of CQ are safe for use in cell culture, higher, and potentially toxic, concentrations are required to enhance transfection efficiency in vivo. To avoid toxicity, one potential approach is incorporation of the lysosomotropic compounds with nanocarriers that provide localized release inside the lysosomal compartments.

Gene therapy approach often requires prolonged periods for effective treatment of genetic-based diseases; therefore, sustained availability of nucleic acids may be required [6]. For RNA interference-based cancer treatments, prolonged therapeutic nucleic acid delivery to the cancerous cells increases the chance of successful gene knockdown [7]. In recent years, sustained gene release based on scaffold-mediated gene delivery has provided a more effective strategy for in vivo and clinical gene therapy. Sustained delivery of genes which are complexed with nanocarriers might benefit from continuous exposures to the therapeutic genes as well as facilitated delivery of genes by a nanocarrier.

Microsphere-encapsulation of nucleic acids serves as a simple approach for controlled release [8, 9]. Alginate, a natural-derived biopolymer, is commonly used to microencapsulate cells and biomolecules due to its excellent characteristics such as bio-inertness, fast in situ gelation, and tunable properties [10]. It has been reported that microencapsulation of viral vectors in alginate microbeads could result in enhanced transfection efficacy while decreasing immune responses [11]. However, most of the reported studies microencapsulated naked nucleic acids into the alginate microbeads.

Graphene and its derivatives have been extensively studied as drug and gene delivery carriers in recent years [12]. To improve graphene's properties such as solubility, dispersibility, biocompatibility, cell penetration capability, and capacity for gene/drug loading, various covalent or nano-covalent surface functionalization approaches have been tried [13]. We have previously investigated graphene oxide (GO) functionalization with octaarginine (R8) as a novel nanocarrier for gene delivery application [14]. The transfection potential of R8-functionalized GO was improved by co-conjugation of polyethylene glycol (PEG)-diamine. It was demonstrated that the optimization of the PEG: R8 molar ratio would lead to a desirable plasmid and siRNA delivery vehicle for breast cancer cell lines [15]. Folic acid (FA) has been identified as a potential ligand that targets cancers which overexpress folate receptors. FA functionalization could improve the nanocarrier's potential to deliver prognostic and therapeutic cargos [12].

In the current study, the possibility of FA, R8, and PEG-diamine multifunctionalization of GO (GPP) to achieve targeted transfection potential is explored. Loading and co-delivery of chloroquine (CQ) along with nucleic acid could potentially enhance systemic treatment and consequently provide safer condition for in vivo gene therapy. The rationale is that localized release of CQ inside the lysosome could lower pH locally without affecting other compartments. This would minimize the effective doses, increasing safety and transfection efficacy. Therefore, microencapsulated of designed multifunctional nanocarrier complexed with siRNA in alginate microbeads containing CQ to facilitate sustained gene release in cancer treatments. To achieve more prolonged release as well as increasing biostability of the alginate against fast in vivo dissolution, the microbeads were coated with poly L-lysine (PLL) through electrostatic interaction [16, 17]. Layer-by-layer-coated alginate microbeads (APA) were investigated for the sustained release of siRNA/nanocarrier.

## 2. Materials and Methods

### 2.1. Materials

All the reagents used for synthesis and functionalization of GO nanosheets were purchased from Sigma-Aldrich, Canada, except otherwise indicated. The MCF-7 and MBA-MD 231 breast cancer cell lines were purchased from American Type Culture Collection (ATCC, Manassas, USA). Fetal bovine serum (FBS) and Dulbecco's modified eagle's medium (DMEM) were purchased from Invitrogen (Montreal, CA). The MTS viability assay kit was obtained from Promega (Madison, USA). The FITC-labeled scrambled siRNA and all western blot assay reagents were supplied by Santa Cruz Biotechnology (Ontario, CA). AllStars Hs Cell Death Control siRNA was supplied from QIAGEN (Montreal, CA). LysoBrite® and Hoechst 33342 staining reagents were bought from AAT Bioquest® (Montreal, CA) and GIBCO®-Life Technology (Montreal, CA). PEG-diamine, chloroquine, EDC, NHS, glutaraldehyde, FITC, DMSO, FA, alginate, poly-L-lysine, and ethidium bromide (EtBr) were supplied by Sigma (Montreal, CA). Ocataarginine (R8) cell penetrating peptide was bought from Abbiotec™ (CA, Montreal).

### 2.2. Preparation of Functionalized Nanocarrier

#### 2.2.1. Preparation of Graphene Oxide Nanosheets (GON)

Graphene oxide nanosheets (GON) were synthesized using Hummers' method and further oxidized and exfoliated, as previously described [18]. R8 and PEG-diamine were introduced to GON (GPP) based on the protocol described in our previous work [15]. Briefly, 1 mg GON dispersed in 5 mL MES buffer under a mild bath sonication followed by addition of EDC and NHS (10 mg of each) to activate COOH groups. The R8 (1 *μ*mol) and PEG (10 *μ*mol) were dissolved in PBS and added to the activated GON under sonication (1 h) and stirred in 4°C for 24 hrs. The mixture was washed by filter centrifugation (30 KD) to obtain GPP which was resuspended in water and kept at 4°C.

#### 2.2.2. Folic Acid Functionalization of Nanocarrier (GPPF)

Folic acid (FA) was introduced to GPP nanocarriers as depicted in Schematic 1: (a) by physical adsorption of FA on GPP surface through *π*-*π* interaction (GPP/FA), (b) covalent conjugation of FA to free amine groups of GPP (GPP-FA), and (c) simultaneous covalent co-conjugation of FA with R8 and PEG-diamine (GPPF). In all three strategies, the GO: FA ratio was similar.

*(1) GPP/FA*. FA was dissolved in DMSO to obtain 10 mg/mL concentration. In the dark, 200 *μ*L of FA solution was added to 2 mL of GPP solution (0.5 mg/mL) under sonication (1 h). The mixture was stirred at 4°C for 24 hrs. Unbounded FA was removed using the filter centrifugation washing steps.

*(2) GPP-FA*. GPP was dispersed in the MES buffer to reach a concentration of 0.5 mg/mL. 200 *μ*L of FA solution in DMSO (10 mg/mL) was diluted in 1 mL of MES buffer followed by adding 2 mg EDC and NHS to activate the FA carboxyl groups. After 30 min, FA solution was added to GPP solution under sonication to react with amine groups of R8 and PEG-diamine. The mixture was stirred overnight at 4°C to induce covalent bonding between carboxyl groups of FA and residual amine functional groups of GPP surface. The final GPP-FA was washed out using several filter centrifugations steps to remove extra reactants.

*(3) GPPF*. As described, the carboxyl-functionalized GO was activated using EDC/NHS. After replacing MES buffer with PBS, 200 *μ*L of FA solution in DMSO (10 mg/mL) was added to GO solution along with R8 (1 *μ*mol) and PEG (10 *μ*mol). After 1 h sonication, the mixture was stirred at 4°C for 24 hrs, and unreacted materials were removed by filter centrifugation.

### 2.3. FA-Functionalized Nanocarrier Characterization

To evaluate the successful loading of FA onto the GPP carrier, three types of formulations (GPPF) were characterized by UV-Vis spectroscopy (Cary-100 bio, Varian Inc.) to record spectra of each specimen suspended in deionized (DI) water. Zeta potential analyzer (Brookhaven Instruments Corporation, USA) was used to measure the size and surface charges of specimens. For estimation of FA loading onto the GPP, free FA (unloaded) was used as control for UV-Vis measurement. UV-Vis absorption of FA solutions at different concentrations (12.5-250 *μ*g/mL in MES buffer) was recorded at 281 nm to provide a standard calibration curve. After FA immobilization, the solutions were centrifuged and the concentration of unreacted FA in the supernatants was estimated using the calibration curve. The loading of FA was estimated by subtracting the free FA from the initial FA concentration. Finally, the dispersibility of GPP nanocarriers after FA functionalization was measured in PBS and cell culture media containing serum proteins after 48-hr incubation at 4°C using DLS analysis [19].

### 2.4. Cellular Uptake of FA-Functionalized Nanocarrier

The effect of FA functionalization on cellular uptake efficacy was examined by transfection of MCF-7 cells with the optimized FA-functionalized GPP. The optimized FA-functionalized GPP was labeled with FITC (SI.1) to be screened under confocal fluorescent microscopy (Carl Zeiss, Jena, Germany). Cells were cultured in 96-well plate with a density of 1 × 10^4^ cells per well (24 hrs before transfection). Then, 100 ng of FITC-GPPF were drop-wise added to the cells. After 1 and 4 hrs, the medium was removed, and transfected cells were washed with PBS, fixed with 0.3% glutaraldehyde for 5 min, and stained with Hoechst® (0.5 g/mL in PBS) for 15 min. The lysosomes were also stained using LysoBrite® (0.1 g/mL in PBS) for 5 min. The staining media was replaced with PBS, and confocal microscopy was performed using a ×60 oil-immersion objectives and postprocessed using Zeiss LSM 510 software.

The cellular uptake efficiency was calculated by measuring fluorescence intensity using a microplate reader (excitation: 480 nm, emission: 520 nm) after 24-hr incubation. The internalization efficacy of the nanocarriers was expressed as relative fluorescence intensity of GPPF-FITC rather than GPP-FITC [15].

Cell internalization mechanism of FA-functionalized nanocarrier was also analyzed by transmission electron microscopy (TEM) [20]. Briefly, MCF-7 cells at density of 10^5^cells/well were cultured in a 6-well plate Petri dish for 24 hrs. After removing the culture media and rinsing the cells with PBS, 40 *μ*L of nanocarriers (GPP and GPPF) was dispersed in 1 mL culture media and was added to the wells and incubated for 1 and 4 hrs. The cells were rinsed and treated by 500 *μ*L trypsin for 5 min. The aspirated cells were centrifuged at 1500 rpm to form a cell pellet that was transferred to a microtube containing 500 *μ*L glutaraldehyde. After 15-min incubation at 4 C, the pellet was centrifuged at 5000 rpm for 8 min, fixed, and embedded in epoxy resin. The pellets were cut in 60 nm (ultrathin sections) using an ultramicrotome. The ultrathin sections were transferred to the copper grid and stained with 5% uranyl acetate. The copper grids were observed by TEM (Philips169 CM200) at 100 kV.

### 2.5. Loading of Chloroquine (CQ) on Nanocarrier (GPPF/CQ)

Before loading of CQ, in order to optimize the amount of loaded drug, MCF-7 cell viability was evaluated in the presence of a range of different molarities (10-250 *μ*M) of CQ. The cytotoxicity of CQ was evaluated by 3-(4,5-dimethylthiazol-2-yl)-5-(3-carboxymethoxyphenyl)-2-(4-sulfophenyl)-2H-tetrazolium (MTS) assay using the Cell Titer 96® Aqueous Non-Radioactive Cell Proliferation MTS Assay kit (Promega, Madison, USA). Briefly, triplicates of 1 × 10^4^ cells were seeded in a 96-well plate and cultured for 24 hrs followed by treatment with CQ (under light protection condition) at 10-250 *μ*g/mL (37°C) for 24 and 48 hrs. The MTS assay was performed based on the manufacturer protocol. The viability of cells was calculated as the percentage of viable cells to untreated cells as control. Furthermore, morphology of cells was observed by optical inverted microscope. Furthermore, to estimate the maximum CQ adsorption on the nanocarrier, CQ fluorescence quenching in the presence of different concentrations of GPPF (0-400 *μ*g/mL) was investigated. 100 *μ*L of CQ solution (20 *μ*M in carbonate-bicarbonate buffer, pH 11) was added to a 96-well plate under light protected condition. Subsequently, GPPF nanocarrier solution (in carbonate-bicarbonate buffer, pH 11) was added to each well. Well containing CQ (without nanocarriers) and well containing nanocarriers in the absence of CQ were considered as negative control and blank sample, respectively. The plate was incubated for 24 hrs under light protection (room temperature, shaking 150 rpm). Then, the fluorescence intensity of CQ was measured by a fluorescent microplate reader (310 nm excitation and 390 nm exposure). CQ quenching percent (Q%) was reported based on the following equation:

(1) $$\begin{array}{c} \%=100\times \frac{E_{\text{test}}-E_{\text{blank}}}{E_{\text{control}}}, \end{array}$$

where *E*_test_, *E*_blank_, and *E*_control_ were considered as exposure intensity of test, blank, and control sample, respectively. GPPF nanosheets were dispersed in carbonate-bicarbonate buffer (pH 11) with a concentration of 100 *μ*g/mL. CQ solution (1 mg/mL in DI water) protected from light was added to the GPPF solution with a final concentration of 10 *μ*M. The CQ/nanocarrier mixture was incubated at room temperature for 24 hrs. To remove the extra CQ, the nanocarrier was washed with carbonate-bicarbonate buffer. The CQ loaded nanocarrier (GPPF/CQ) was kept at 4°C in the buffer.

### 2.6. CQ Release Kinetics

CQ release in different pH conditions was evaluated via gel electrophoresis analysis [21]. GPPF/CQ was incubated in 500 *μ*L DI water (37 °C, 24 hrs), with the pH adjusted to 4.5, 7.4, and 11 using NaOH 1 M or HCl 1 M. Then, 12 *μ*L of each sample with 3 *μ*L of sucrose 40% w/v (as loading buffer) was loaded in an agarose gel (0.8% in TBE buffer) along with different concentrations of CQ solution (12 *μ*L CQ + 3 *μ*L sucrose) in acidic condition (pH 4.5). Electrophoresis was run for 30 min at 90 V with 40% sucrose as loading buffer. The gel was detected by an automatic digital gel image analysis system (Hercules, CA). Finally, CQ band intensities were quantified with the image analysis software ImageJ® (quantitative densitometry tool) to estimate the amount of released CQ.

### 2.7. Lysosomotropic Effect

The lysosomotropic effect of the delivered CQ by GPPF nanocarrier was evaluated using confocal fluorescent microscopy. In order to transfer FITC-labeled siRNA into MCF-7 cells by GPP/CQ nanocarrier, 100 ng FITC-siRNA was mixed with the nanocarrier solution (N/P ratio = 10) and left for 30 min at room temperature to form a complex. The complexes were then added to 10^4^ cells and incubated for 4 hrs. The medium was removed, and cells were washed with PBS. Subsequently, the transfected cells were fixed with 0.3% glutaraldehyde for 5 min and stained with Hoechst® (0.5 g/mL in PBS) for 15 min. The lysosomes were also stained using LysoBrite® (0.1 g/mL in PBS) for 5 min. The staining media was replaced with PBS, and the cells were imaged by confocal microscope. The CQ-treated cells (15 *μ*M, without nanocarrier) and nontreated cells were used as positive and negative controls, respectively.

### 2.8. Cell Death Induction by siRNA Delivery

Cell death control siRNA (cd-siRNA) was transfected against MCF-7 breast cancer cells by GPP, GPPF, and GPPF/CQ at N/P ratio of 10. The cells were seeded into a 96-well plate with a density of 1 × 10^4^ cells/well. After 24 hrs, an appropriate amount of the samples were mixed with 75 ng cd-siRNA in a 100 *μ*L medium without serum and left for 30 min at room temperature. The cells were washed with PBS, and the mixture was drop-wise added on the cells for transfection. The cell death caused by siRNA delivery was evaluated using MTS viability assay after 72 hrs of incubation.

### 2.9. Western Blot Analysis

The knock down of c-Myc protein expression for MDA-MB 231 breast cancer cells was inspected by western blot analysis after 72 hrs of transfection. Total proteins were extracted from nanocarrier/siRNA-transfected cells, resolved electrophoretically on 12% sodium dodecyl sulfate (SDS)–polyacrylamide gel and transferred on polyvinylidene fluoride (PVDF) membranes. The membranes were blocked overnight at 4°C and probed with rabbit primary polyclonal anti-C-Myc (1: 900 dilution) and GAPDH *β* (1: 900 dilution) antibodies at 25°C for 2 hrs. After incubation for 50 min with horseradish peroxidase (HRP)-labeled goat anti-rabbit secondary antibodies, the proteins were detected using ECL reagent. Bands intensities were compared relative to the nontransfected cells used as control.

### 2.10. Layer-by-Layer Microencapsulation of Nanocarrier

Encapsulation of nanocarrier loaded by scrambled siRNA in alginate/poly-L-lysine (Alg/PLL) microcapsule was performed using an Inotech® Encapsulator IER-20 (Inotech Biosystems International, Rockville, MD, USA) with a nozzle of 200 𝜇m diameter under sterile conditions [22]. Briefly, 100 ng siRNA was mixed with the nanocarrier solution (N/P ratio =10) and left for 30 min at room temperature to be complexed. The complexes were suspended in an autoclaved sodium alginate solution (2% in DI water) containing 4.5% (v/v) NaCl (0.1 M) solution. The alginate/nanocarrier suspension was loaded into a microencapsulator device syringe and inject into calcium chloride bath (0.1 M) while gently stirring. The droplets were allowed to form a gel for 15 min. The alginate gel microcapsules were collected with a filter and washed with NaCl (0.1 M) solution. The alginate microcapsules were dispersed into PLL solution (0.1% w/v) for 20 min, collected with filter paper and washed with NaCl solution. Alg/PLL microcapsules were subsequently dispersed into alginate solution (0.1% w/v) for 20 min. Finally, Alg/PLL/Alg (APA) microcapsules were collected by filter paper, washed with NaCl and keep at 4°C.

### 2.11. Characterization of Alg/PLL/Alg (APA) Microcapsules

The structure of as-prepared microcapsules containing the nanocarrier/gene complexes was analyzed using optical microscopy. Furthermore, scanning electron microscopy (SEM) was used to examine morphology of APA microcapsules. The microcapsules were freeze-dried for 24 hrs, sputter coated with gold/palladium using a Hummer VII sputter coater (Anatech Ltd., Alexandria, VA, USA), and observed with a JEOLT330A SEM at 10 kV.

### 2.12. Gene Release Analysis

5 mL of microencapsulated nanocarrier/siRNA were incubated in 5 mL of PBS while gently shaking at 37 °C. After each time point 50 *μ*L of media was removed and replaced with 50 *μ*L of fresh PBS. The released nanocarrier/pEGFP complexes were analyzed using gel electrophoresis [23]. Briefly, 15 *μ*L of collected sample was mixed with 2 *μ*L of loading dye (6×) and loaded on the agarose gel (1% in TBE buffer) containing EtBr (0.5ug/mL). Naked siRNA was also loaded under similar conditions and used as control. Electrophoresis was run at 100 V for 30 min, and the gels were detected by an automatic digital gel image analysis system (Hercules, CA).

### 2.13. Statistical Analysis

All data are presented as the mean ± SD of at least three experiments. Statistics was calculated with SPSS software (v 17.0; IBM New York, NY, USA) using a two-way ANOVA followed by Tukey's multiple comparison test. Results were considered statistically significant when *p* < 0.05.

## 3. Results and Discussion

### 3.1. FA-Functionalized GPP Characterization

**Figure 1:**
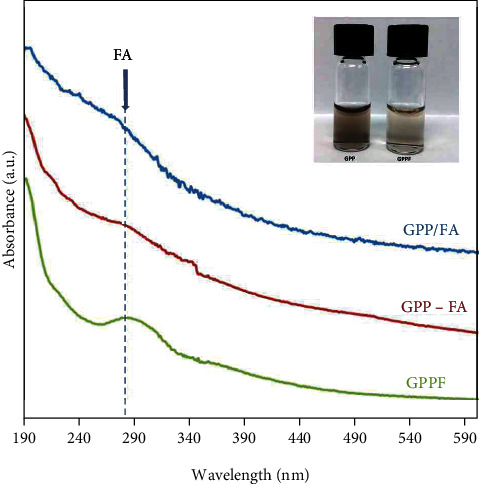
UV-Vis spectra of FA-functionalized GPP via different routes. Inset shows the color change of GPP solution after FA functionalization.

The GPP nanocarrier was functionalized with FA via three different mechanisms as shown in Schematic 1. Considering GPP functional groups and aromatic rings in basal planes of graphene nanosheets, FA could be immobilized on the nanocarrier via covalent bonding [24] and physical *π*-*π* stacking [25]. UV-Vis spectra of all FA-functionalized formulations showed the characteristic peak at 282 nm which is representative of successful immobilization of FA on the nanocarrier (Figure 1). As it can be seen in the inset of Figure 1, the color of the solution containing FA-functionalized GPP was changed to yellowish brown after FA introduction. Based on the FA calibration curve and measuring nonbonded FA concentration (SI. 2), it was found that a significantly higher amount of FA was incorporated into the GPP-FA formulation rather than GPPF and GPP/FA (Table.  1). This is an indication for a greater efficiency of covalent interaction compared to the physical adsorption of FA molecules via *π*-*π* stacking. Oxidation of graphene to GO as well as GO functionalization with R8 and PEG-diamine may result in losing graphitic domains which limit *π*-*π* interactions. Furthermore, steric hindrances of other ligands including R8 and PEG could also interfere with the physical *π*-*π* stacking. However, comparing the two covalent FA-immobilized formulations elucidated that there was no significant difference in the amount of FA bonding via amine groups or carboxyl groups.

The results of DLS and zeta potential measurements for different FA-functionalized GPP formulations are listed in Table 2. As shown in Table 2, FA functionalization of GPP carrier significantly decreased their zeta values. However, the charge of nanocarriers remained positive. Although FA was intended to enhance targeting ability of the nanocarriers, positive charge density is also necessary for interactions with the cell membrane. The mean effective diameter for FA-functionalized formulations showed an increase except for GPPF. The amine groups of GPP were subjected to the covalent reaction with FA; therefore, a large decrease of zeta value and increase of mean size was expected.

Since hydrocolloidal stability of the nanocarrier is an essential requirement for successful gene transfection, nanocarrier stability in PBS and serum supplemented culture media was evaluated. As shown in Table 3, GPP stability was considerably affected by FA immobilization in the GPP-FA formulation. This was anticipated considering the zeta potential decrease of this sample. The noncovalent functionalization of FA also resulted in some instability for GPP nanocarriers as a significant size increase was detected in both PBS and DMEM media. However, simultaneous co-conjugation of GO with FA along with R8 and PEG provided suitable hydrocolloidal stability, with no aggregation or precipitation of the nanocarrier.

Considering FA immobilization efficacy, the nanocarrier charge and size values, as well as hydrocolloidal stability, GPPF was selected as the best formulation for further functionalization and characterization.

### 3.2. GPPF Cellular Uptake

**Figure 2:**
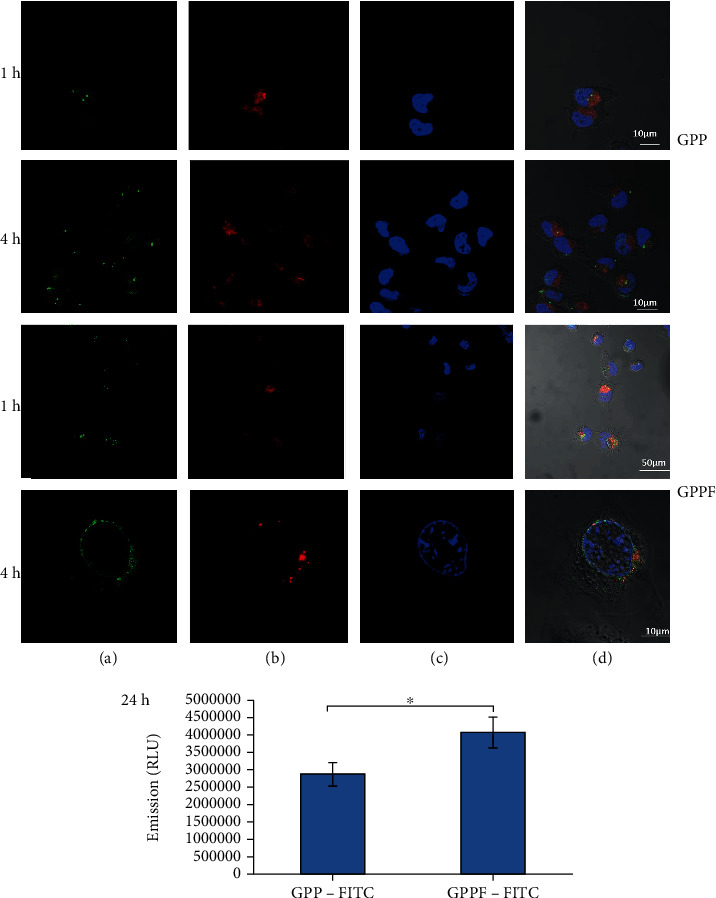
Confocal images of GPP and GPPF nanocarriers uptake by MCF-7 cells after 1 and 4 hrs of incubation. The images show FITC-labeled nanocarrier (a), lysosome staining (b), nucleus staining with Hochest3342 (c), and merged images (d). Quantitative internalization efficacy of GPP and GPPF nanocarrier after 24 hrs was represented in the lower panel.

An efficient gene knockdown or expression is dependent on delivery of the carrier/gene complex across the cell membrane [26]. As targeted cellular uptake could enhance transfection efficacy, the FITC-labeled GPPF cellular uptake was investigated prior to gene knockdown experiment. MCF-7 is a FA-receptor positive breast cancer cell line [27]; therefore, to evaluate the effect of FA immobilization on the GPP cellular uptake, FITC-GPPF and FITC-GPP were exposed to MCF-7 cells for 1 and 4 hrs. After 1-h incubation, the FITC-GPPF nanocarriers were internalized by the cells as indicated by abundant green dots inside the lysosomal compartments which were absent in cells treated with FITC-GPP (Figure 2). After 4 hrs of incubation, FITC-GPPF showed much higher internalization and release into the cytoplasm in comparison with the FITC-GPP nanocarrier. The quantified internalization efficacy after 24 hrs also showed a significant improvement for the GPPF-FITC formulation relative to FITC-GPP (Figure 2). As mentioned earlier, FA has a specific receptor on the cell membrane which is internalized by receptor-mediated endocytosis. Therefore, FA functionalization of the nanocarriers along with R8 could facilitate their cellular entrance and enhance the cellular uptake. It has also been reported that FA conjugation of chitosan-functionalized GO could improve MCF-7 cellular uptake via receptor-mediated endocytosis [28].

**Figure 3:**
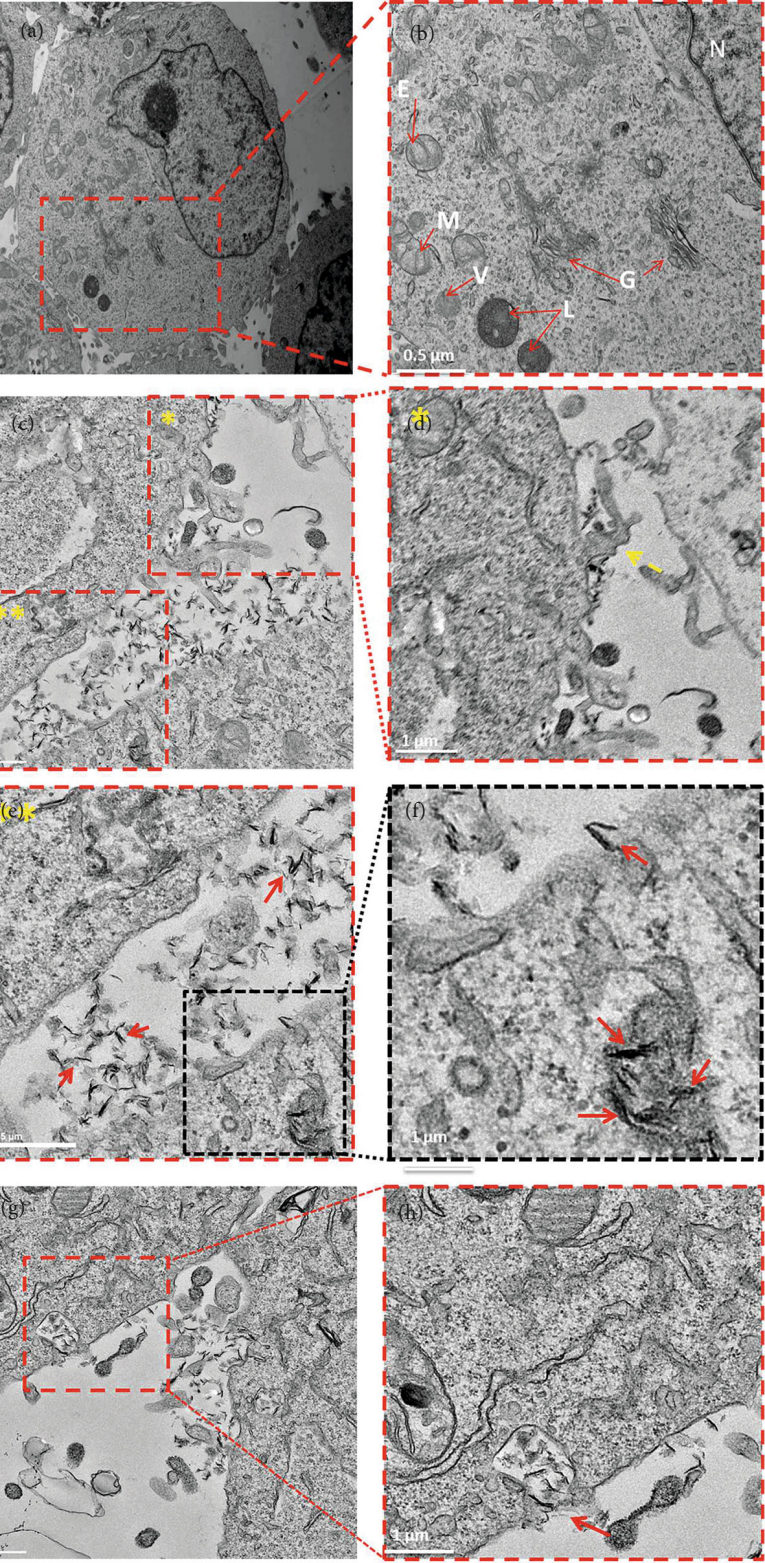
TEM images of MCF-7 cells incubated with 40 *μ*L of GPPF nanocarrier (50 *μ*g/mL). Intracellular compartment in control cells were labeled, where E, M, V, L, G, and N represent endosome compartment, mitochondria, vacuole, lysosomes, Golgi apparatus, and nucleus (a & b). Nanocarriers accumulation and localization adjacent to the cell membrane (c & e). Membrane blebbing and protrusion (arrows) to internalize the nanocarriers via possible macropinocytosis (c & f). Accumulation of nanocarriers inside lysosomal compartments (e & f). Membrane pitting (arrows) to internalize the nanocarriers via possible receptor-mediated endocytosis (g & h).

For more detailed investigation, the ultrastructure of the MCF-7 cells treated with GPPF was observed by TEM. The cell compartments were clearly detectable on TEM analysis (Figures 3(a) and 3(b)). It was found that the GPPF had entered the cell and was located near the MCF-7 cell surface (Figures 3(c) and 3(e)). Furthermore, GPPF trafficking across the cell membrane and accumulation in intracellular vacuoles were observed. Interestingly, the cell membrane showed some pitting which can be indicative of receptor-mediated endocytosis (Figures 3(g) and 3(h)) [29] similar to that previously reported for the folate receptor [30]. It is also reported by Huang et al. that FA-functionalized graphene nanoparticles are mainly internalized by receptor-mediated endocytosis [29]. However, some GPPF nanoparticles showed perpendicular orientation and apparent penetration through the cell membrane (Figure 3(f)). In addition to receptor-mediated endocytosis, macropinocytosis was also detectable (Figures 3(c) and 3(d)) [29]. Macropinocytosis usually involves blebbing and protrusion of the plasma membrane to wrap around the nanoparticle [29, 31]. The mechanism of the nanoparticles internalization into the cells is mainly affected by their surface chemistry, size, and functionalities [32]. It is likely that FA incorporated into the structure of GPPF favors receptor-mediated endocytosis, while R8 may favor macropinocytosis, since it is known that arginine-based cell penetration peptides can favor macropinocytotic cell entry [33]. However, macropinocytosis induction has been shown to depend on the peptide density [34]. In one study, an increase in the concentration of conjugated R8 altered the cellular uptake mechanism of silica nanoparticles to favor macropinocytosis [34]. Further observations of the GPPF-treated MCF-7 cells after 4-hr incubation revealed that most of the nanoparticles were internalized into the cells and accumulated inside the lysosomal compartment or released into the cytoplasmic space (Figure 3(f)), which is in agreement with the confocal microscopic images (Figure 1). TEM observations confirmed that not only did the receptor-mediated endocytosis facilitate the nanocarriers' uptake, but also that induced macropinocytosis by R8 ligands may play a role in cellular uptake. The precise density of immobilized R8 and FA on the nanocarrier structure is not easy to control; therefore, it is possible that a synergistic effect of the ligands could affect the cellular uptake mechanism. The surface charge and size are also among the other important parameters that could direct the cellular uptake of functionalized graphene sheets [35].

### 3.3. CQ-Loaded GPPF Characterization

In gene therapy, the escape of delivered cargo from degradative lysosomal compartments is a crucial part of the function of an efficient nanocarrier. The co-delivery of CQ along with genes could improve the lysosomal escape potential of the nanocarrier. The effect of CQ on the cells is highly dose-dependent. High concentrations of CQ often leads to apoptosis, while lower concentration may enhance lysosomal escape potential without causing cell death [36].

**Figure 4:**
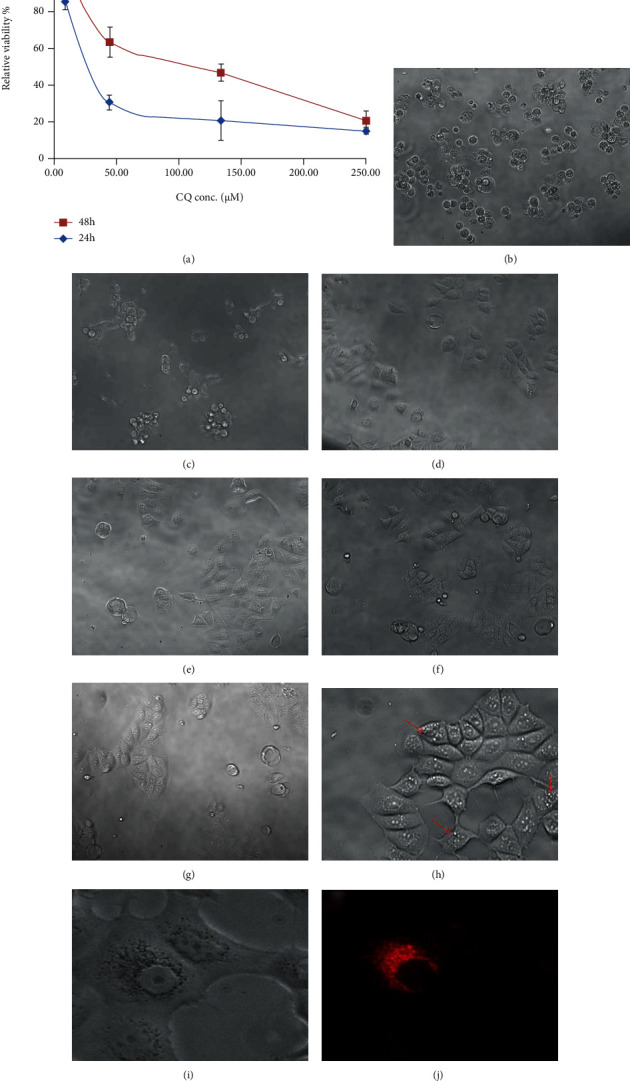
(a) Relative cell viability of MCF-7 cells incubated with different concentration of CQ after 24 and 48 hrs. (b–g) Optical microscopy images of MCF-7 cells treated by 250, 133, 50, 10, 1 and 0 *μ*M of CQ (after 24 hrs). (h) Phase contrast image of MCF-7 cells vacuolation under treatment of 10 *μ*M CQ. (i and j) Optical and fluorescent microscopy images of lysosomal hypertrophy effect observed in MCF-7 cells under treatment of 10 *μ*M CQ. Lysosomal compartments stained in red with LysoBrite®.

The MTS results demonstrated that CQ concentration of higher than 30 *μ*M dramatically affected cell viability after 24 and 48 hrs of incubation (Figure 4(a)). A concentration of lower than 10 *μ*M resulted in a relative viability of more than 80%. In addition, morphological observation of the cells under light microscopy revealed that increasing CQ concentration from 1 *μ*M to 50 *μ*M did not significantly affect their morphology (Figures 4(b)–4(g)), whereas at 133 and 250 *μ*M, significant morphological changes was detected, as reported by other studies [36]. At 10 *μ*M, phase contrast and fluorescent observations (Figures 4(h)–4(j)) of MCF-7 cells stained with LysoBrite ® showed a major increase in vacuolation as a result of lysosomal hypertrophy [37], suggesting effects on the lysosome that could favor nanocarrier escape. Therefore, 10 *μ*M CQ was selected as the optimum loading concentration.

**Figure 5:**
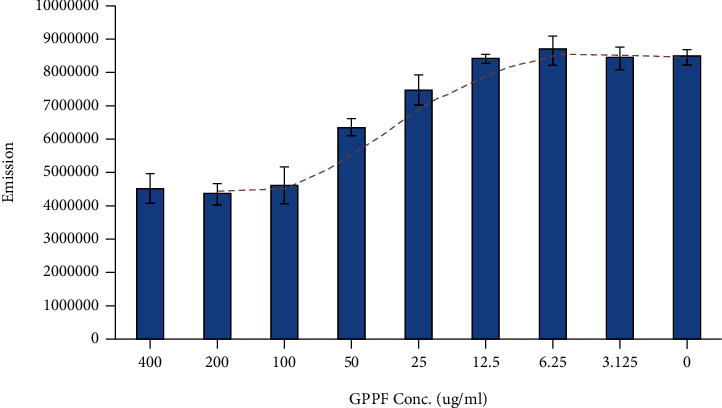
CQ fluorescent quenching effect in the presence of different concentrations of GPPF.

CQ has been reported to be immobilized on carbon nanotubes by Sanz et al. [21]. A similar strategy was used to immobilize CQ on the graphene nanosheets via *π*-*π* stacking. Since CQ interaction with graphene via *π*-*π* stacking may result in fluorescent quenching, the fluorescent quenching was assessed to evaluate the saturation value of the CQ on the GPPF [21]. As shown in Figure 5, an increase in GPPF concentration up to 100 *μ*g/mL led to gradual quenching of CQ, while for 200 and 400 *μ*g/mL of GPPF, the quenching reached a plateau. It was established that at 100 *μ*g/mL, the maximum amount of CQ was immobilized on the GPPF via physical *π*-*π* interaction. It was demonstrated that for 15 *μ*M of CQ, 25-100 *μ*g/mL of GPPF would provide the quenching condition. Considering 100 *μ*g/mL GPPF as upper threshold of quenching and the amount of CQ in each well (15 *μ*M), it was estimated that 0.01 *μ*M of CQ was immobilized on 100 ng of GPPF.

**Figure 6:**
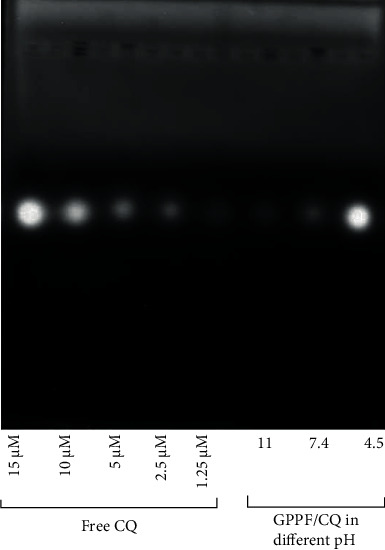
Gel electrophoresis assay showing the release of CQ from the nanocarrier under a variety of pH conditions (11, 7.4, and 4.5). Different concentrations of free CQ (1.25-15 *μ*M) were simultaneously loaded to prepare a calibration cure (SI-7).

Gel electrophoresis was used to investigate CQ release under a range of different pH's [21]. CQ was released in at acidic pH, while in the basic condition its release was negligible (Figure 6). CQ is a weak amphiphilic base that exists in both charged and uncharged forms in acidic (or neutral) and basic pH, respectively [5]. Therefore, in the acidic condition, the cationic form of CQ demonstrates higher solubility leading to a faster release from the GPPF/CQ complex. The amount of released CQ was estimated by Image J® calibration wizard (SI.3). As estimated, around 95.3% of CQ was released under the acidic condition, while 3.2% and 9.6% of CQ were released at basic and neutral condition, respectively. The results support that there is localized pH sensitive release of CQ from GPPF after entrapment in the lysosomal compartments.

**Figure 7:**
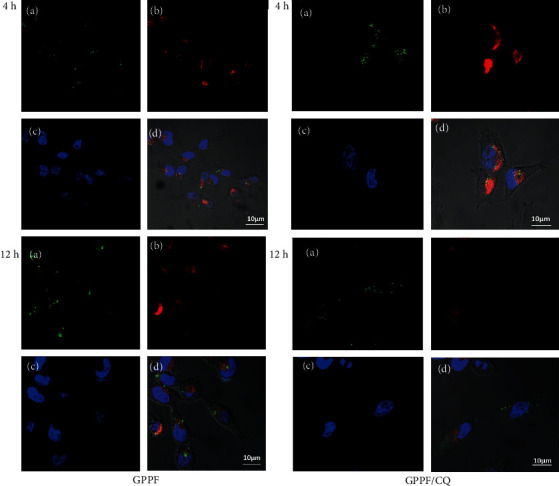
Confocal images illustrating the uptake and localization of GPPF and CQ-loaded nanocarriers (GPPF/CQ) by MDA-MB 231 cells after 4 and 12 hrs of incubation. The images show siRNA-labeled FITC (a), lysosome staining (b), nucleus staining with Hochest3342 (c), and merged image (d).

The lysosomotropic effect of the delivered CQ was investigated under confocal microscopy by staining of the lysosomal compartments using LysoBrite®. Since the MCF-7 cells have FA receptor, to distinguish between the CQ and FA, MDA-MB 231 breast cancer cells were treated by siRNA-FITC instead of MCF-7. As depicted in Figure 7, after 4-hr incubation, cells transfected with CQ-loaded GPPF showed a significant increase in lysosomal swelling and vacuolation. However, the amounts of green dots which represent accumulated siRNA-FITC in the lysosomal compartments were similar in GPPF/CQ and GPPF-treated cells. After 12-hr incubation, the amount of released siRNA-FITC found in the cytoplasm was considerably higher in GPPF/CQ treated cells. These data suggest that the incorporation of CQ and its localized release inside the lysosomal compartments accelerated the cargo discharge into cytoplasm via enhanced lysosomal instability.

### 3.4. siRNA Transfection

**Figure 8:**
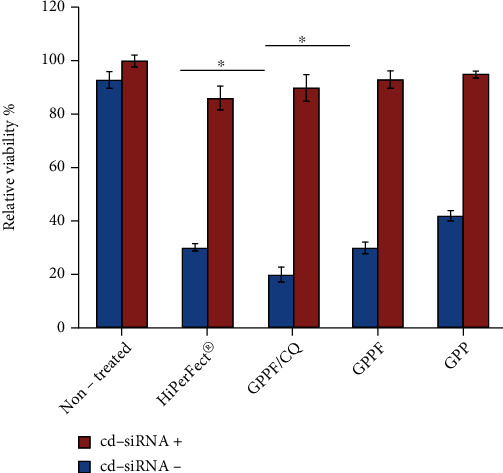
Histogram representing cell viability of different formulations with similar concentrations (50 *μ*g/mL) ∗ represents *p* < 0.05.

To evaluate transfection ability of the multifunctionalized GO and subsequent gene knockdown, AllStars® cell death control siRNA, a blend of highly potent siRNA against genes that are vital for cell survival, was used [38]. Cells were transfected by cell death siRNA (cd-siRNA)-loaded GPPF/CQ, GPPF, and GPP nanocarriers, and induced cell death was quantitatively measured by MTS assay. The results showed that functionalization of GPP nanocarriers complexed with cd-siRNA induced considerable cell death after 72 hrs of incubation. The FA immobilization on GPP- and CQ-loaded GPPF showed 61% and 75% decreases in cell viability, respectively. Based on the cellular uptake results, it was anticipated that the uptake as well as localized release of lysosomotropic agent in endosomes would enhance the siRNA transfection capacity.  Figure 8 shows that delivery of bare nanocarrier into the cells did not significantly affect the MCF-7 cell viability; however, cd-siRNA transfection into the cells resulted in a significant decrease in cell viability for all the formulations.

While some attempts have been made to use co-delivery of CQ to enhance gene transfection efficacy, there is no report that investigate CQ loading on graphene nanosheet gene carriers. Bhattarai et al. synthesized polycation-modified mesoporous silica nanoparticles that were capable of simultaneous delivery of CQ and nucleic acids [39]. They showed a significant increase in transfection and silencing activity of the complexes compared with bare silica nanoparticles. Hartono et al. developed a novel silica-based nanocarrier to deliver PLK1-siRNA into KHOS cells. They loaded CQ on the mesoporous silica in order to facilitate endosomal escape. Their results confirmed that incorporation of CQ into the nanocarrier could increase biological function of the delivered siRNA. However, they did not investigate gene knockdown. Almost all of the studies that utilized CQ treatment before or during gene transfection have used a concentration of CQ around 100-300 *μ*M [40]. It should be noted that such condition cannot be employed in vivo due to the high systemic toxicity of CQ. Here, the effective concentration of the CQ was 10 *μ*M, which is considerably lower than the toxic concentrations used in previous studies. Successful and efficient delivery of CQ with the GPPF nanocarriers resulting in its localized release could provide safer and more effective treatment for in vivo and clinical applications. The cells can be safely transfected by the cd-siRNA-loaded nanocarrier which could lead to successful gene silencing.

### 3.5. Functional Gene Knockdown

**Figure 9:**
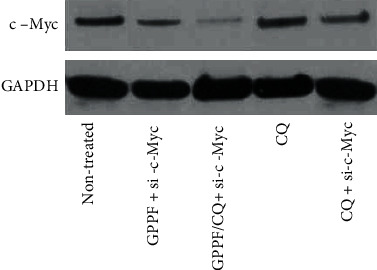
Western blot analysis of c-Myc (up) and GAPDH (down) of protein expression in si-c-Myc treated MDA-MB 231 cells.

c-Myc, a multifunctional gene, plays a key role in most cellular functions including replication, growth, metabolism, differentiation, and apoptosis [41, 42]. Upregulation of c-Myc is detectable in a wide range of cancer. Knockdown of the c-Myc gene may increase the probability of controlling cell metastatic behavior [43]. PrMDA-MB 231 breast cancer cells were transfected and c-Myc knockdown assayed by Western blot. While western blot analysis showed that c-Myc siRNA delivered by GPPF nanocarrier was downregulated c-Myc protein expression in MDA-MB 231 cells, c-Myc knockdown was significantly higher in GPPF/CQ (Figure 9). Cells treated only with CQ did not exhibit an effective c-Myc protein downregulation. As expected, siRNA-treated cells in the presence of similar concentration of CQ showed some degree of c-Myc knocking down; however, it was less than in GPPF/CQ-siRNA-treated cells. Consequently, CQ-loaded nanocarriers appear to provide greater inhibition compared to GPPF alone. This is likely due to localized release of CQ inside lysosomal compartments.

### 3.6. Microencapsulation of Nanocarriers and Gene-Sustained Release

The layer-by-layer-coated alginate microbeads (APA) were prepared and optimized as previously described [44]. Before encapsulation, the nanocarrier/siRNA complexes were analyzed by DLS and zeta charge. As expected, the charge of the nanocarriers decreased after complexing with siRNA, while the net charge remained positive enough to interact with negatively charged alginate chains. The complex size was decreased as compared to the bare nanocarrier which is an indication of siRNA condensation through electrostatic interaction.

**Figure 10:**
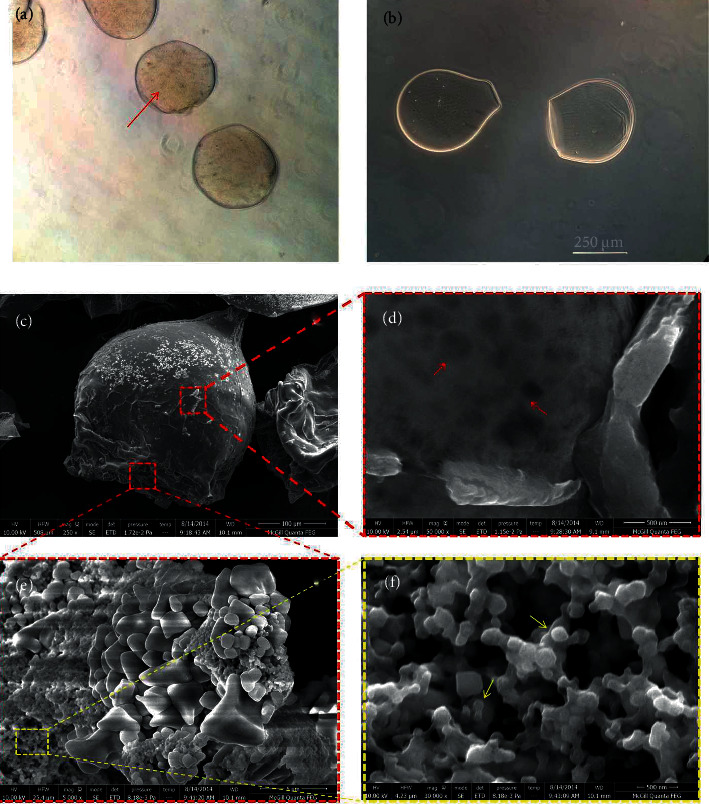
Optical microscope images of alginate microcapsules loaded by nanocarrier/gene complexes (a) compared to nonloaded microcapsules (b). SEM images of APA microcapsules loaded by nanocarrier/gene complexes (c–f). The arrows show encapsulated nanocarrier/gene complexes.

Figures 10(a) and 10(b) show optical microscopic images of the alginate core before and after the encapsulation of gene/nanocarrier complexes. Almost all complex-free microbeads showed a dumbbell-like shape, whereas the complex encapsulated samples had spherical morphology with irregular edges. This could originate from enhanced viscosity of the alginate solution resulting from the addition of the complexes and subsequent electrostatic interactions [45]. The size of the hydrated alginate core was around 250-300 *μ*m, and the complexes were detectable inside the microbead resembling a brownish aggregate (red arrows).

The SEM observation revealed that the freeze-dried microbeads were uniform in size and shape (Figures 10(c)–10(f)). However, the cross-section of the beads showed a porous structure. Interestingly, the complexes were detectable both on the surface and in cross-sectional view (arrows) where their size was consistent with the DLS measurement (SI.4).

**Figure 11:**
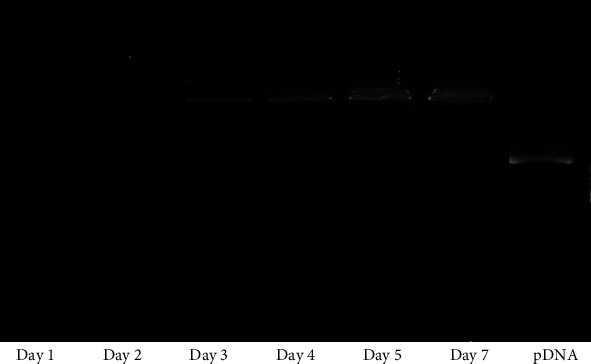
Gel retardation assay representing the release of gene/nanocarriers complexes from APA microcapsules over time.

The sustained release of complexes from APA microbeads was investigated by an agar gel retardation assay where naked siRNA was loaded as a control. The siRNA was detected by ethidium bromide staining [23]. As depicted in Figure 11, retention of the complexes in the wells was the lowest in day 1. Retention increased by time up to 7 days. In contrast, naked siRNA (without nanocarrier) was not well retained and moved across the gel. This indicates that the released siRNA was in bounded form to a nanocarrier which resulted in its retention in the well, whereas the unbounded siRNA could move freely in the gel. These results are consistent with the previous study of Paul et al. that utilized a sustained release of VEGF for cardiac regeneration application and reported a similar gene release kinetic from a hydrogel matrix [23]. It is believed that the alginate-based layer by layer encapsulation could successfully provide a desirable sustained release profile for the siRNA-bound nanocarrier during the first week.

## 4. Summary and Conclusion

The potential of PEG/R8/FA multifunctionalized GO loaded by CQ as siRNA-based gene delivery nanocarrier was investigated. The FA was successfully coconjugated to the nanocarrier by step-wise conjugation (physical and chemical), and the efficacy of FA immobilization was optimized. The optimized nanocarrier formulation (GPPF) contained 0.068 *μ*mol/mg of FA with a charge of +31.97 mV and size of 224 nm which remained stable in biological solution. The addition of FA to the R8/PEG-functionalized nanocarrier significantly enhanced the rate of MCF-7 cellular internalization via receptor-mediated endocytosis as shown by TEM analysis. However, the macropinocytosis pathway was also induced as a result of R8 functionality in the GPPF formulation. Incorporation of CQ at an optimized concentration of 10 *μ*M via *π*-*π* stacking empowered GPPF nanocarrier to escape from the lysosomal compartment due to the lysosomotropic effect of CQ. Furthermore, the pH-dependent release of CQ (95.3% in pH 4.5) from nanocarriers provided more efficiency and safety than free CQ treatment. The MTS and western blot analyses demonstrated that cell death control siRNA and anti-c-Myc siRNA that delivered by GPPF/CQ were highly functional after successful transfection of the cells. The alginate-based layer-by-layer encapsulation of gene/carrier with average size of 250-300 *μ*m successfully provided a prolonged release pattern of the nanocarrier bound gene during 7 days. It is believed that the synthesized and optimized multifunctional nanocarrier has great potential to be utilized as a gene carrier for potential targeted cancer therapy. Additionally, the sustained release of gene loaded GPPF/CQ from APA microbeads could improve efficacy of a wide range of in vivo and clinical gene therapy applications.

## Figures and Tables

**Scheme 1:**
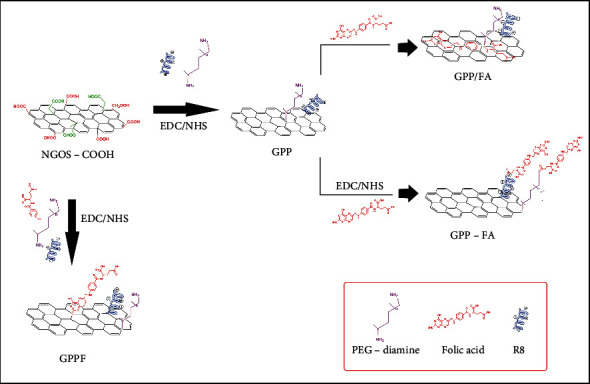
Three different routes that were utilized to functionalize GPP nanocarriers with FA.

**Table 1:** The loaded amount of FA on GPP nanocarrier using three different routes.

Formulation	GPP/FA	GPP-FA	GPPF
Bonded FA (*μ*g)	0.015 ± 0.006	0.035 ± 0.004	0.030 ± 0.008
FA (*μ*mole) bonded	0.043	0.079	0.068

**Table 2:** Zeta potential and particle size analysis of FA-functionalized GPP compared to GPP.

Sample	Zeta potential value(mv)	Particle size analysis	
		Effective diameter (nm)	PDI
GPP/FA	+20 ± 2.4	266 ± 2.8	0.35 ± 0.008
GPP-FA	+12 ± 1	321 ± 8.8	0.38 ± 0.001
GPPF	+31.97 ± 2.1	224 ± 4.6	0.21 ± 0.007
GPP	+40.97 ± 1.05	252 ± 2.0	0.11 ± 0.007

**Table 3:** Hydrocolloidal stability assessment of FA-functionalized GPP formulations during incubation in DMEM and water. ∗*p* value<0.05 (1 compared to 48 hrs).

Sample	Effective diameter (nm) in DMEM		Effective diameter (nm) in deionized water	
	After 1 h incubation	After 48-h incubation	After 1-h incubation	After 48-h incubation
GPP/FA	314 ± 7.3	418 ± 9.1∗	270 ± 3.6	398 ± 5.5∗
GPP-FA	388 ± 3.3	612 ± 11.2∗	325 ± 8.8	754 ± 21∗
GPPF	237 ± 2.1	243 ± 5.5	229 ± 4.6	238 ± 4.1

## Data Availability

All data would be available on request.

## Supplementary Materials

Supplementary Materials SI-1. FITC labeling of nanocarrier. SI-2. Folic acid calibration curve. SI. 3 CQ Calibration curve. SI.4 Zeta potential and particle size analysis.
