# Supplementary material for: Microencapsulated Multifunctionalized Graphene Oxide Equipped with Chloroquine for Efficient and Sustained siRNA Delivery
Source: Biomed Res Int. 2022 Apr 6;2022:5866361. doi: 10.1155/2022/5866361 (PMC9034959; doi:10.1155/2022/5866361)
Supplement: Supplementary Materials — SI-1. FITC labeling of nanocarrier. SI-2. Folic acid calibration curve. SI. 3 CQ Calibration curve. SI.4 Zeta potential and particle size analysis. [file 5866361.f1.docx]

**Supplementary information**


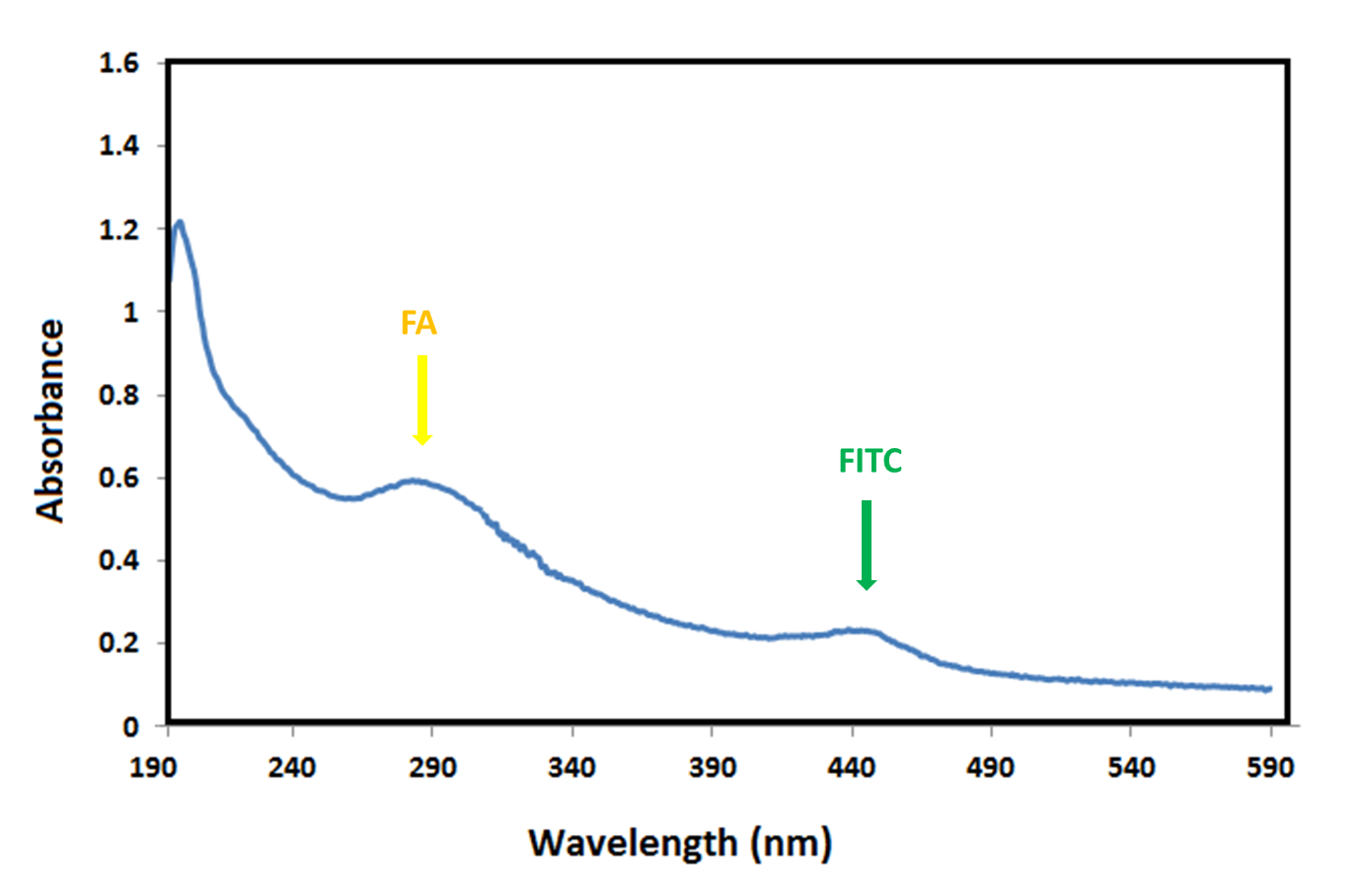


**SI-1. FITC labeling of nanocarrier**

In order to be able to trace the nanocarriers inside the cells, they were labeled with FITC according to the supplier protocol. Briefly, FITC was dissolved in DMSO in concentration of 5 mM. 0.1 mg of nano-carrier was dispersed in 1 ml of carbonate-bicarbonate buffer (pH 9.2) under bath sonication. 10 µL of FITC was added to nano-carrier dispersion and shaked for 2 hrs at room temperature. The FITC-labeled nano-carrier was separated from the free FITC by several washing steps. The Uv-vis spectrum of FITC-labeled GPPF nanocarrier is depicted above. Both FITC and FA characteristic peaks were also visible.


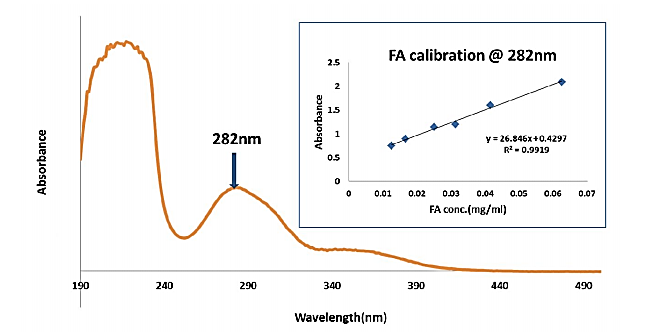


**SI-2. Folic acid calibration curve**

Uv-vis spectrum and calibration curve of FA were prepared based on absorption at 282 nm for various FA concentrations (12.5-250 μg/mL in MES buffer).


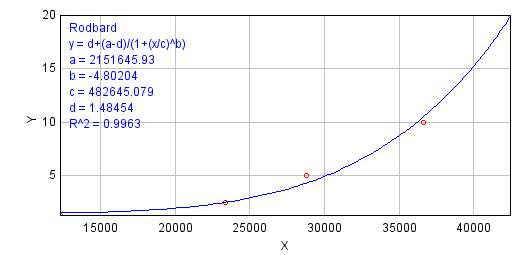


| **4.5** | **7.4** | **11** | **Incubation pH** |
| --- | --- | --- | --- |
| 95.3 | 9.6 | 3.2 | **Estimated Release %** |

**SI. 3 CQ Calibration curve**

Calibration curve of CQ concentration (X) versus illumination density (Y), estimated by Image J^®^ calibration wizard. The concentration of released CQ at different pH was estimated based on the optical densitometry of gel electrophoresis image, compared to the prepared calibration curve.

**SI.4 zeta potential and particle size analysis**

Zeta potential and particle size analyses of functionalized nanocarriers complexed with plasmid compared to bare functionalized nanocarriers (GPPF/CQ)

| **Sample** | **Zeta potential value (mv)** | **Particle size analysis** | |
| --- | --- | --- | --- |
|  |  | **Effective diameter (nm)** | **PDI** |
| **GPPF/CQ** | +35.97±2.05 | 245±3.0 | 0.18±0.01 |
| **GPPF/CQ + pEGFP** | +22.43±2.05 | 218±4.15 | 0.21±0.005 |
